# Genetic Variations Affecting Serum Carcinoembryonic Antigen Levels and Status of Regional Lymph Nodes in Patients with Sporadic Colorectal Cancer from Southern China

**DOI:** 10.1371/journal.pone.0097923

**Published:** 2014-06-18

**Authors:** Yu Liang, Weizhong Tang, Tiqiang Huang, Yong Gao, Aihua Tan, Xiaobo Yang, Haiying Zhang, Yanling Hu, Xue Qin, Shan Li, Shijun Zhang, Linjian Mo, Zhenjia Liang, Deyi Shi, Zhang Huang, Yingyong Guan, Jicheng Zhou, Cheryl Winkler, Stephen J. O'Brien, Jianfeng Xu, Zengnan Mo, Tao Peng

**Affiliations:** 1 Department of Hepatobiliary Surgery, First Affiliated Hospital of Guangxi Medical University, Nanning, Guangxi, People's Republic of China; 2 Department of Anal and colorectal Surgery, First Affiliated Hospital of Guangxi Medical University, Nanning, Guangxi, People's Republic of China; 3 Center for Genomic and Personalized Medicine, Guangxi Medical University, Nanning, Guangxi, People's Republic of China; 4 Department of Occupational Health and Environmental Health, School of Public Health, Guangxi Medical University, Nanning, Guangxi, People's Republic of China; 5 Medical Scientific Research Center, Guangxi Medical University, Nanning, Guangxi, People's Republic of China; 6 Department of Clinical Laboratory, First Affiliated Hospital of Guangxi Medical University, Nanning, Guangxi, People's Republic of China; 7 Institute of Urology and Nephrology, the First Affiliated Hospital of Guangxi Medical University, Nanning, Guangxi, People's Republic of China; 8 Medical Examination Center, Fangchenggang First People's Hospital, Fangchenggang, Guangxi, People's Republic of China; 9 Medical Examination Center, Guigang First People's Hospital, Guigang, Guangxi, People's Republic of China; 10 Medical Examination Center, Yulin First People's Hospital, Yulin, Guangxi, People's Republic of China; 11 Department of Hematology, First Affiliated Hospital of Guangxi Medical University, Nanning, Guangxi, People's Republic of China; 12 Molecular Genetics Epidemiology Sec., Frederick Nat. Lab for Cancer Research, National Cancer Institute, NIH, Frederick, Maryland, United States of America; 13 Laboratory of Genomic Diversity, National Cancer Institute, NIH, Frederick, Maryland, United States of America; 14 Theodosius Dobzhansky Center for Genome Bioinformatics, St. Petersburg State University, St. Petersburg, Russia; 15 Oceanographic Center, Nova Southeastern University, Ft. Lauderdale, Florida, United States of America; 16 Center for Cancer Genomics, Wake Forest University School of Medicine, Winston-Salem, North Carolina, United States of America; Shanghai Jiao Tong University School of Medicine, China

## Abstract

**Background:**

Serum carcinoembryonic antigen (sCEA) level might be an indicator of disease. Indeed, an elevated sCEA level is a prognostic factor in colorectal cancer (CRC) patients. However, the genetic determinants of sCEA level in healthy and CRC population remains unclear. Thus we investigated the genetic markers associated with elevated serum sCEA level in these two populations and its clinical implications.

**Methods and Findings:**

Genome-wide association study (GWAS) was conducted in a cohort study with 4,346 healthy male adults using the Illumina Omni 1 M chip. Candidate SNPs associated with elevated sCEA levels were validated in 194 CRC patients on ABI Taqman platform. Eight candidate SNPs were validated in CRC patients. The rs1047781 (chr19- FUT2) (A/T) was associated with elevated sCEA levels, and rs8176746 (chr9- ABO) was associated with the regional lymph metastasis in the CRC patients. The preoperative sCEA level was a risk factor for tumor recurrence in 5 years after operation (OR = 1.427, 95% CI: 1.005∼1.843, P = 0.006). It was also one of the risk factors for regional lymph node metastasis (OR = 2.266, 95% CI: 1.196∼4.293, P = 0.012). The sCEA level in rs1047781-T carriers was higher than that in the A carriers in CRC patients without lymph node metastasis (P = 0.006). The regional lymph node metastasis in patients with homozygote AA of rs8176746 was more common than that in the heterozygote AG carriers (P = 0.022). In addition, rs1047781-AT and TT CRC patients exhibited a worse disease-free survival than AA genotype carriers (P = 0.023).

**Conclusions:**

We found candidate SNPs associated with elevated sCEA levels in both healthy males and CRC population. Rs1047781 (chr19- FUT2) may be the susceptible locus for recurrence of CRC in a population from Southern China.

## Introduction

Colorectal cancer (CRC) is the fourth most common cancer in males and the third most common in females worldwide. It accounts for an estimated 1.2 million new cancer cases and over 630,000 cancer deaths per year [1]. Incidence and mortality of CRC rose rapidly in China since the 1990s [2]. Sporadic CRC is the most common that occurs in the absence of family history, generally affects older population, and usually presents as an isolated colon or rectal lesion. Curative surgery is the most important approach of treatment. However, 5-year survival after resection is merely 60.8 % in China. Approximately 50% of these patients would undergo tumor relapse and die [3]. The prognosis was affected by factors such as cigarette smoking, age, gender and race, duration of symptoms, presence of bowel obstruction, tumor location, blood transfusion and the quality of surgical intervention [4].

Carcinoembryonic antigen (CEA) level might be elevated in the serum of people with non-neoplastic diseases, malignant tumors or cancers. Thus an elevated CEA level is an independent prognostic factor in CRC patients regardless of its Duke's stage and histological grade [5]. In the healthy population, serum CEA levels could be affected by environmental and genetic factors such as age, smoking, drug, disease, gender and race [6]–[8]. However, in patients with CRC, additional mechanisms determining serum CEA level remain unclear.

Genome mutations associated with the elevated CEA level in CRC is present in the tumor itself, so such mutations could not be served as a genetic marker for CEA level for diagnostic purposes [9]. Therefore, this study was conducted in two phases: at phase one, a genome-wide association study was conducted to identify the loci associated with the serum level of CEA in healthy Chinese men from a cohort that participated in the Fangchenggang Area Male Health and Examination Survey (FAMHES) (this part has been merged with other cohorts and partly published elsewhere [10], [11]). At phase two, the association between candidate SNPs found from phase one GWAS and preoperative CEA levels and prognosis of patients with CRC was analyzed. Based on this design, we tried to explore in this study the association between polymorphisms and the prognosis of CRC after radical operation on account of susceptible loci associated with elevated sCEA level.

## Methods

### Phase one study

A genome-wide association study was conducted to identify the loci associated with the serum level of CEA in healthy Chinese men from FAMHES cohort (The experiment and results were described in Text S1, Tables S1, S2, S3, and Figure S1).

### Phase two study

#### Patients with sporadic colorectal cancer

One hundred and ninety-four colorectal carcinoma patients who met the criteria for enrollment were recruited from patients undergoing curative resection between March 2008 and February 2012 at the Department of Anal and Colorectal Surgery, 1^st^ Affiliated Hospital of Guangxi Medical University. All tumors in the study were adenocarcinoma, and the pathological diagnoses were confirmed independently by two expert gastrointestinal pathologists. All patients were absent of symptoms such as severe bleeding, bowel obstruction or infection. None of them had any preoperative radio-chemotherapy. All patients were treated with postoperative chemotherapy protocol based on NCCN Clinic Practice Guideline in Oncology. Written informed consent was required from all patients. They were followed up to 51 months, and by the end there were 57 patient with tumor recurrence (median survival: 48 months; range: 7∼62 months). This study was approved by the ethics committee of Guangxi Medical University. Baseline characteristics of participants were summarized in Table S4.

#### Genotyping of candidate SNPs in CRC patients

Nine candidate SNPs from the 25 SNPs that reached genome-wide significance were selected for genotyping in CRC patients: rs8176746 (chr9- ABO), rs1047781 (chr19-FUT2), rs3760775 (chr19-FUT6), rs441810 (chr21-FAM3B), rs12608544 (chr19-DBP), rs3786749 (chr19- SULT2B1), rs2071699 (chr19- FUT1), rs507666 (chr9- ABO) and rs687289 (chr9- ABO).

DNA was extracted from the colon mucous membranes using a DNA Extraction Kit (Tiangen, China). PCR platform (STEPONE PLUS ∧TM, Applied Biosystem. Foster city, CA) was used to conduct allele's genotyping. Automatic SNP genotyping was run by StepOne Plus ∧TM version 2.2 system (Applied Biosystem. Foster city, CA); the quality control criteria used were the same as that in GWAS. The details of experiment are described in Text S1 and Table5.

#### Chemotherapy

According to the risk for post-operation recurrence, some patients at stage IIand all stage III patients received at least one cycle of Oxalipatin, Fluorouracil, and Leucovorin (FOLFOX regiment) as adjuvant treatment in our study. Patients at age ≥70 years did not receive adjuvant treatment. Adverse effects were assessed using the Common Toxicity Criteria of National Cancer Institute version 2.0. Chemotherapy was stopped when there was cardiac or neurocerebellar adverse effects or grade 3 or 4 allergic reaction.

#### Follow-up

Patients in our study were assessed every six months after operation. The recurrence was determined on the basis of physical examination, chest radiography, abdominal ultrasonography or computer tomography, colonoscopy, CEA level detection, and if necessary, cytologic analysis or biopsy. The cut-off date of the analysis was June 20, 2013; duration of follow-up was defined as the number of months from operation to the cut-off date. The disease-free survival was measured from the time of operation to the recurrence of tumor.

#### Statistical analyses

Nonparametric statistics was used in univariate analysis. Multivariable models were constructed by Logistic regression analysis. Kaplan-Meier estimators were applied in calculating survival function; the log-rank test was used to determine the survival differences among individual groups. The SPSS v.17.0 program was used for analysis and a P value <0.05 was considered statistically significant.

## Results

### Factors affecting sCEA levels in healthy population

In General Linear Regression model, Age (Beta = 0.003, P<0.0001), total quantity of cigarettes (Beta = 0.004, P<0.001) and BMI (Beta = -0.005, P = 0.006) was significantly associated with CEA level (**Table 1**). R^2^ of this model was 0.07 (F =  51.834, ANOVA).

**Table 1:** Multiple linear regression analysis of the factors affecting sCEA levels in FAMHES participants.

Variables	B	Beta	p	95% C.I for B	Adjusted R^2^
(constants)	1.365	0.043	<0.0001	0.19∼0.357	0.070
Age	0.003	0.147	<0.0001	0∼0.004	
Total quantity of cigarettes	0.004	0.175	<0.0001	0∼0.005	
BMI	-0.005	-0.06	0.006	-0.008∼0	

B: unstandardized coefficients; Beta: standardized coefficients. Data of CEA values had been log-transformed for normal distribution. Regression model (F-value of ANOVA: 51.384; p<0.01).

### Genotyping in CRC patients

The candidate SNPs used to genotype for 194 CRC patients with adenocarcinoma and the genotyping results were listed in Table S6.

### Risk factors for elevated CEA levels in CRC patients

The univariate analyses revealed that the preoperative CEA level was significantly different by status of regional lymph nodes and distant organ metastases (positive vs. negative: 5.36 vs. 3.41 ng/ml; P = 0.011; 11.87 vs. 3.60 ng/ml; P = 0.033. respectively). The preoperative CEA level was also not associated with factors such as age, gender, cigarette smoking, alcohol drinking, tumor size and cell differentiation (**Table 2**).

**Table 2:** Univariate analysis of factors affecting the preoperative CEA levels in CRC patients.

Variables		n	sCEA Level (ng/ml)^1^	*P* value
Age (yrs.)	≥60	93	3.30	0.077
	<60	96	4.34	
Gender	Male	117	3.67	0.689
	Female	72	4.19	
Cigarette smoking	Yes	45	4.29	0.606
	No	145	3.49	
Alcohol drinking	Yes	41	4.61	0.202
	No	149	3.49	
Tumor size(cm)^2^	≥5	94	3.52	0.194
	<5	94	4.66	
Tumor Differentiation	Well	9	3.43	0.719
	Moderately	153	3.80	
	Poorly	21	3.49	
Distant organ metastasis	Yes	16	11.87	0.033
	no	174	3.60	
Regional lymph nodes metastasis	yes	77	5.36	0.011
	no	112	3.41	
Depth of tumor invasion	I	1	-	0.129
	II	44	2.26	
	III	60	3.54	
	IV	1	-	

1 The CEA level was represented by median.

2 The tumor size was represented by the max diameter of primary tumor. The patients with regional lymph nodes metastases or distant metastases were excluded in this analysis.

In patients without regional lymph nodes metastasis, subgroups with rs1047781 (chr19- FUT2) AT and TT allele had a significantly higher CEA level than the AA carriers (AA:1.73 ng/ml, AT: 3.49 ng/ml and TT: 4.11 ng/ml; P = 0.006). However, the difference was not significant in the CRC patients with lymph node or distant organ metastasis (AA vs. AT: P = 0.686, AA vs. TT: P = 0.202, AT vs. TT: P = 0.139, respectively). The J-T test showed that the TT allelic subgroup had a higher sCEA level while the AA had the lowest (P = 0.011). Other candidate SNPs were not associated with CEA levels in CRC patients (**Table 3**). For rs1047781 (chr19- FUT2), the allelic genotype TT was associated with the highest sCEA level, and the difference in the sCEA level between allele T carriers and homozygote AA was significantly different (AA vs. AT: P = 0.008; AA vs. TT: P = 0.006; AT vs. TT: P = 0.787; respectively) (**Table 4**).

**Table 3:** Association between candidate SNPs and preoperative CEA levels in CRC patients stratified by status of regional lymph nodes metastasis.^1^

			Metastasis (yes)				Metastasis (no)			
SNP	allele	n	sCEA levels^2^	*P*	*P*	n	sCEA levels^2^	*P*	*P*	*P*
gene			(ng/ml)	(sCEA)	(St.J-T^3^)		(ng/ml)	(sCEA)	(St.J-T^3^)	(metastasis)
rs8176746	AA	46	5.83	0.179	0.222	66	2.85	0.705	0.557	0.022
*ABO*	AG	18	3.16			33	2.65			
	GG	1	-			1	-			
rs1047781	AA	24	6.39	0.360	0.369	34	1.73	0.006	0.010	0.753
*FUT2*	AT	38	8.29			48	3.49			
	TT	15	3.43			23	4.11			
rs3760775	GG	33	5.23	0.186	0.161	49	2.85	0.750	0.466	0.279
*FUT6*	GT	22	3.67			45	2.85			
	TT	13	17.98			11	3.80			
rs12608544	AA	8	5.83	0.947	0.761	19	2.69	0.040	0.354	0.763
*DBP*	AG	30	5.73			41	4.58			
	GG	30	6.24			45	2.52			
rs3786749	CC	18	6.82	0.687	0.368	35	2.32	0.264	0.912	0.912
*ABO*	CT	19	4.87			28	3.22			
	TT	4	8.13			12	2.36			
rs2071699	AA	3	12.27	0.158	0.437	4	8.69	0.075	0.035	0.814
*FUT1*	AG	24	4.37			43	4.11			
	GG	39	7.24			55	2.52			
rs441810	AA	35	5.36	0.644	0.644	51	3.02	0.261	0.730	0.739
*FAM3B*	AG	21	5.67			37	2.65			
	GG	7	13.5			7	9.66			
rs507666	AA	21	12.2	0.214	-	39	2.52	0.900	-	-
*ABO*	AG	0	-			0	-			
	GG	31	4.11			52	3.19			
rs687289	AA	14	12.86	0.269	0.165	15	2.52	0.419	0.517	0.398
*ABO*	AG	24	6.09			47	2.39			
	GG	25	3.67			42	3.61			

1 The patients with distant metastases were excluded.

2 CEA levels were represented by median.

3 St. J-T statistic was represented using the standard statistic of Jonckheere-Terpstra test.

**Table 4:** Association between RLN metastasis and preoperative sCEA levels (stratified by rs1047781 genotypes).

Metastasis	Allele	(n)	sCEA level^1^	*P*	*P*	Metastasis status		*P*
Type			(ng/ml)	(sCEA)	(St.J-T^2^)	yes(N,%)^4^	no (N, %)	
Regional	AA	24	6.39 (1.10-110.06)	0.287	0.377	24(12.6%)	32(17.8%)	0.784
lymph	AT	38	8.29 (0.50-150000)			37(19.6%)	50(27.8%)	
nodes	TT	15	3.43 (0.92-22.740)			15(9.2%)	26(13.1%)	
metastasis	AA *vs* AT^5^			0.686				0.961
	AA *vs* TT			0.202				0.046
	AT *vs* TT			0.139				<0.001
Distant	AA	4	2.83 (2.10-20.87)	0.304	0.167	4(2.60%)	54(28.2%)	0.860
organ	AT	8	17.53 (0.87-150000)			8(4.04%)	81(43.3%)	
metastasis^3^	TT	4	51.75 (3.85-762.70)			4(1.86%)	37(19.9%)	
	AA *vs* AT			0.368				0.651
	AA *vs* TT			0.114				0.607
	AT *vs* TT			0.808				0.888
Non-	AA	34	1.73 (0.50-21.05)	0.006	0.01			
metastasis	AT	48	3.49 (0.50-282.76)					
	TT	23	4.11 (0.96-21.97)					
	AA *vs* AT			0.008				
	AA *vs* TT			0.006				
	AT *vs* TT			0.787				

1 The values of CEA levels and times were represented by median values, and the range of sCEA level is shown.

2 St. J-T statistic was represented using the standard statistic of Jonckheere-Terpstra test.

3 The distant organs included liver, lung and omentum.

4 The rate of tumor lymph node metastasis was adjusted.

5 The statistical evaluation for CEA levels was performed by Mann-Whitney U test.

### Risk factors for tumor metastasis in CRC patients

The rate of regional lymph node (RLN) metastasis in the allelic subgroup AT was the highest in the three subgroups of rs1047781 (AA: 12.8%, AT: 19.6%, TT: 9.0%; AA vs. AT: P = 0.961, AA vs. TT: P = 0.046, AT vs. TT: P<0.001; respectively). The rates of distant organ metastasis in the three allelic subgroups of rs1047781 were not significantly different (AA vs. AT: P = 0.651; AA vs. TT: P = 0.607; AT vs. TT: P = 0.888, respectively) (**Table 4**). The frequency of RLN metastases was significantly different in the allelic subgroups of rs8176746 (**chr9-ABO**) (P = 0.022).

### Risk factors for disease-free survival in CRC patients

**Figure 1:**
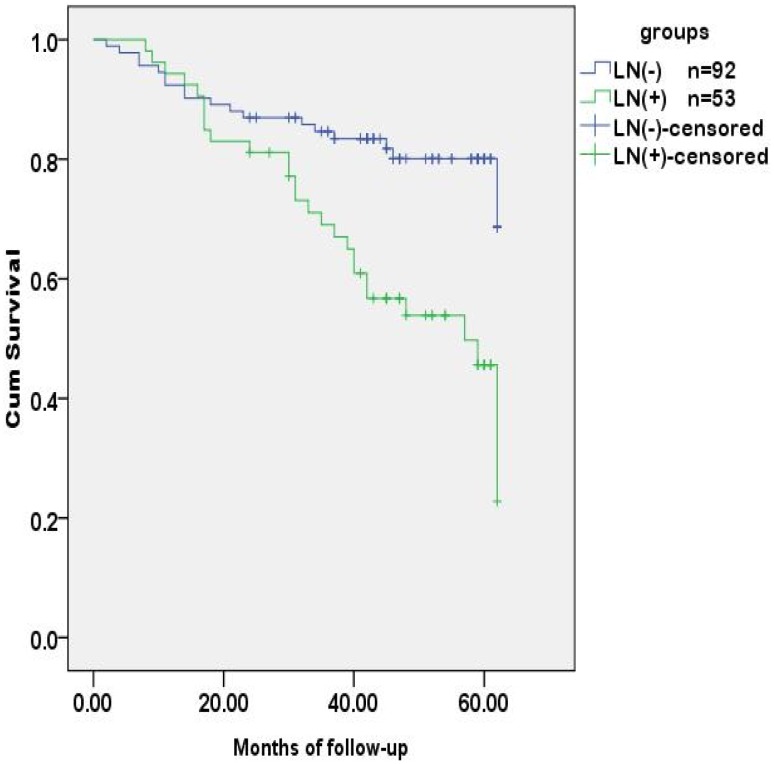
Tumor-free survival analysis of CRC patients stratified by regional lymph nodes. The tumor-free time was different between CRC patients with and without lymph node metastases (*p*<0.001).

Logistic regression analysis revealed that age, preoperative CEA level and T stage (tumor invasion) were risk factors for regional lymph nodes metastasis (**Table 5**). The preoperative CEA level was also the risk factor for tumor recurrence in 5 years after radical operation (**Table 6**). The probability of survival was higher in the group of regional lymph nodes negative than those positive for metastasis, and was lower in the group with a preoperative CEA level of ≥5 ng/ml than those with <5 ng/ml. The differences were statistically significant **(** **Figure 1** **,** **2** **)**. SNPs associated with the CEA level and regional lymph metastasis, rs1047781 (chr19- FUT2) and rs8176746 (**chr9- ABO**), were not the independent contributors to disease-free survival (**Figure 3** **,** **4**). We used the combination of SNPs (rs1047781 and rs8176746) as the genetic marker in survival analysis and the disease-free times was significantly different between rs1047781-AA and rs1047781 (AT+TT) genotype carriers among rs8176746 (AA+AG) carriers (P = 0.023) (Figure **5**). However, the difference was not determined among the rs8176746-GG carriers.

**Figure 2:**
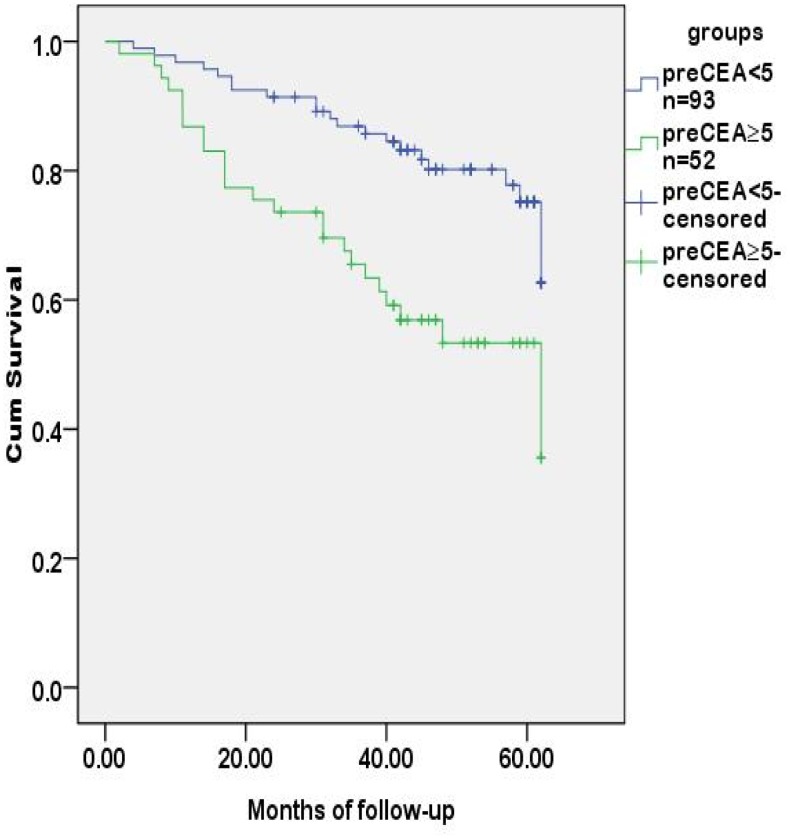
Tumor-free survival analysis of CRC patients stratified by preoperative sCEA levels. The tumor-free time was different between CRC patients with preoperative CEA levels at <5 and ≥5 ng/ml (*p* = 0.001).

**Figure 3:**
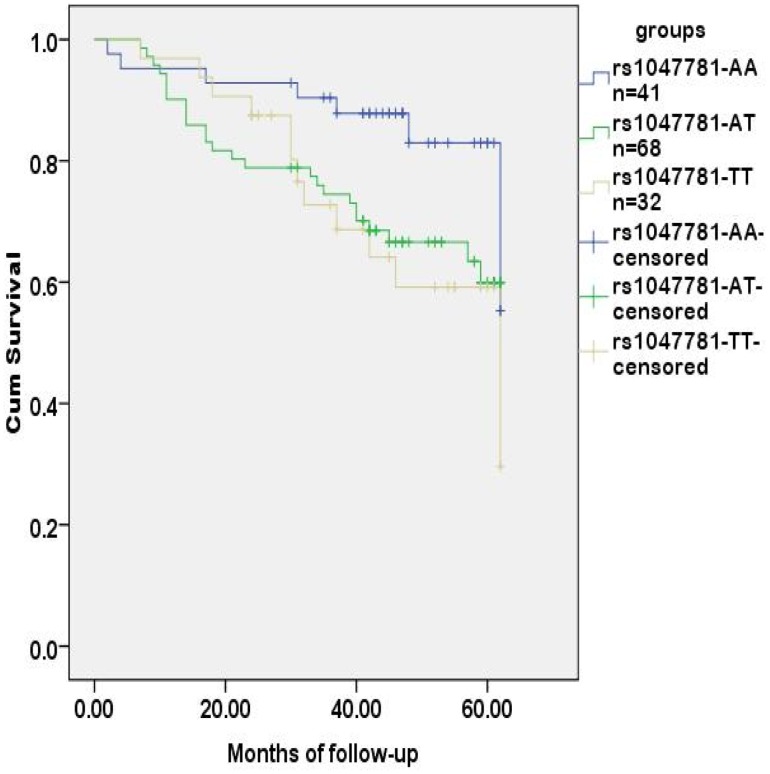
Tumor-free survival analysis of CRC patients stratified by rs1047781. With rs1047781 (chr-19 FUT2), the disease-free time of CRC patients grouped by allelic genotypes (AA, AT and TT) was not significantly different (*p* = 0.066).

**Figure 4:**
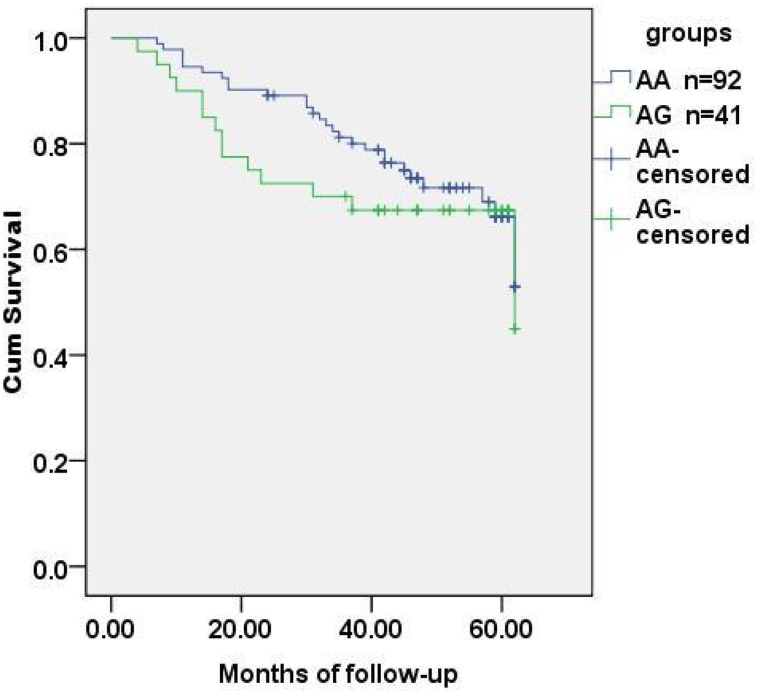
Tumor-free survival analysis of CRC patients stratified by rs8176746 genotypes. With rs8176746 (chr-9 ABO), the disease-free time of CRC patients grouped by allelic genotypes (AA and AG) was not significantly different (*p* = 0.366). Subjects with genotype GG were excluded (n = 2).

**Figure 5:**
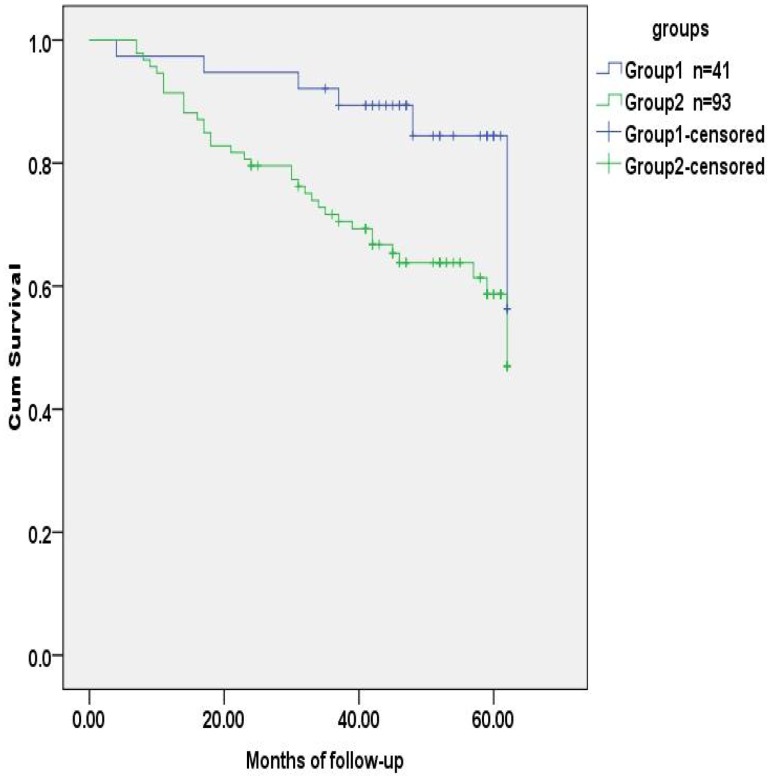
Tumor-free survival analysis of CRC patients stratified by rs1047781+rs8176746 genotypes. Subjects were divided into 2 groups by genetic maker (rs1047781+rs8176746). Group 1 was rs1047781 (AA) +rs8176746 (AA+AG) and group 2 was rs1047781 (AT+TT) +rs8176746 (AA+AG). The disease-free time of subgroups was significantly different (*p* = 0.023).

**Table 5:** Logistic regression analysis of the risk factors influencing regional lymph nodes with tumor metastasis before radical operation.

variables	B	S.E	OR	95%C.I. for OR	*p*
T stage	0.613	0.218	1.847	1.203∼2.835	0.005
Preoperative CEA levels	0.818	0.326	2.266	1.196∼4.293	0.012
Ages	-0.880	0.326	0.415	0.215∼0.801	0.009
constant	-2.198	0.365	0.111		0.001

Variables in the equation included age, gender, tumor size, tumor T stage, preoperative CEA levels and histological grade.

**Table 6:** Logistic regression analysis of risk factors influencing 5-yr recurrence in CRC patients.^1^

Variables	B	S.E	OR	95%C.I. for OR	*p*
Tumor site	-0.492	0.317	0.612	0.329∼1.138	0.121
Macropathological type	-1.122	0.709	0.326	0.081∼1.307	0.114
Cell differentiation	-0.433	1.108	0.649	0.074∼5.696	0.696
Lymph node metastasis	1.103	0.966	3.014	0.454∼20.002	0.253
Chemotherapy	-0.811	1.070	0.414	0.051∼3.373	0.410
Tumor size	1.361	0.933	3.898	0.626∼24.276	0.145
Gender	0.950	0.890	2.586	0.452∼14.801	0.286
Age	0.013	0.03	1.013	0.956∼1.073	0.670
Preoperative CEA levels	0.356	0.131	1.427	1.005∼1.843	0.006
Constant	-0.350	3.457	0.704		0.919

1 The patients with distant organ metastases were excluded.

### ABO genotypes and serum CEA levels

**Figure 6:**
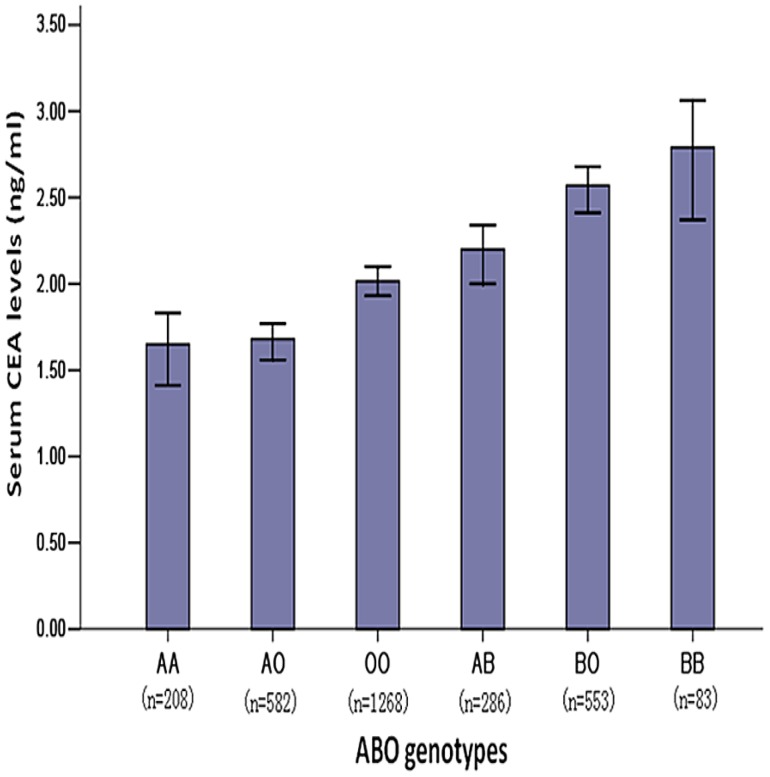
Distribution of serum CEA levels by ABO allelic genotypes in the healthy population. The CEA levels of male FAMHES subjects grouped by ABO groups genotypes were significantly different (p = 1.47E-26).

**Figure 7:**
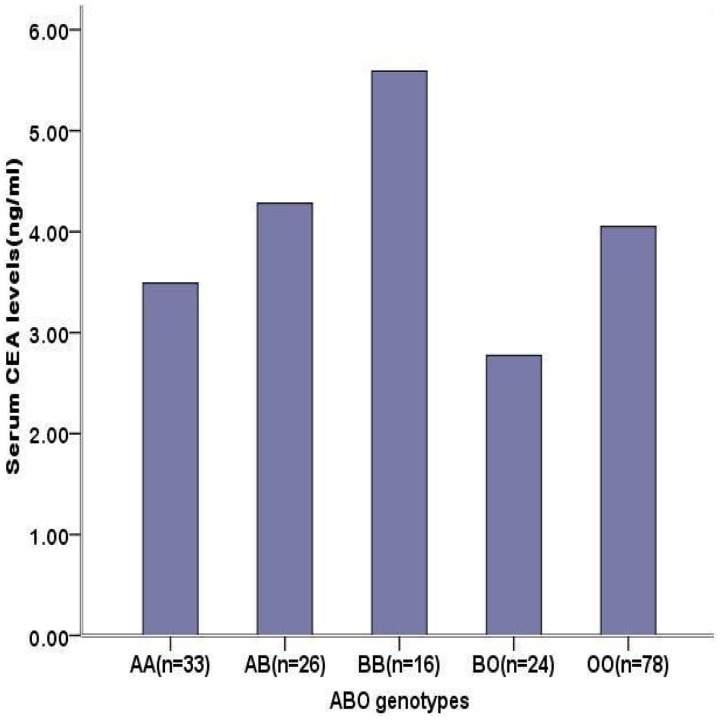
Distribution of serum CEA levels by ABO allelic genotypes in CRC patients. The serum CEA levels of patients with CRC grouped by blood group allelic genotypes were represented by median and there were not significantly different (χ^2^ = 3.63, p = 0.458). Geneotype AO was absent; A1 and A2 were included in A allelic type. The ABO blood type was determined before operation.

The distribution of CEA levels by ABO blood allelic type was significantly different in healthy people (BB>BO>AB>OO>AO>AA, P = 1.47E-26). Although it is not statistically significant (P = 0.458), we also found a similar trend in the CRC patients (B>A) (**Figure 6**, **7**).

## Discussion

### Factors affecting Serum CEA levels in the healthy population

In support of a previous report on healthy volunteers in the United States, we found that cigarette-smoking is associated with CEA levels [12]. Furthermore, when the smoking status is further quantified by the amount of cigarettes (pack per year), a trend in the association between smoking grading and CEA levels was statistically significant. Long term exposure to carcinogens from cigarette-smoking contributes to a variety of malignancies, such as non-small- cell lung cancer, pancreatic cancer and oral cancer [13], [14]. In addition, some studies have reported that an elevated CEA level associated in such malignancies [15]–[17]. Therefore the elevated CEA level might reflect the risk for tumor.

It has been reported that CEA levels increase with aging in France and Netherland populations [18], [19]. However, the trend for higher CEA levels in elders does not necessary correlate with a higher risk for non-neoplastic diseases or cancer [20]. Our finding of the association between elevated CEA level and aging in men from the Fangchenggang area support the notion that age is an independent factor influencing the CEA level in spite of the disparity of population and area. Interestingly, in male smokers, the contribution of age to CEA levels was attenuated.

We found in this study a negative correlation between BMI and CEA level in the healthy male Chinese. It is therefore necessary to consider BMI when conducting GWAS in this population. Similarly, Park JS et al. reported that increased BMI is associated with lower CEA concentrations in CRC patients [21]. Epidemiological studies have also found an association between obesity and colorectal cancer [22]. *In vitro* studies have revealed that the adipocyte could promote the growth of several colon cancer cell lines [23]. Therefore it has been hypothesized that although the CEA is produced by epithelial cells, adipose tissue could affect the secretion of CEA in other tissues *via* various cytokines and adipocytokines.

HBV infection is associated with the risk of hepatocellular carcinoma [24]. However, there is no direct evidence of association between HBV infection and digestive malignancies such as pancreatic cancer and gastrointestinal cancer [25]. In this study, statistical analysis showed that there was no significant difference in the serum CEA levels between groups that are positive or negative for HBsAg. Therefore we did not exclude the samples positive for HBsAg from the GWAS analysis. An increased level of CEA has been found in patients with alcoholic liver diseases or patients with disorders of respiratory and/or gastrointestinal tracts [26]–[28]. However, the association between alcohol-drinking and CEA levels was not conclusive in this study. Therefore, alcohol-drinking is not a justification factor in the GWAS analysis. It has been reported that the serum levels of CEA varied among different ethnic-groups [29]. To exclude ethnic-specific genetic factors, we also limited the number of Han participants in GWAS.

Of note, we found that age, cigarette smoking and obesity to be the epidemiology factors affecting serum CEA levels. Nevertheless, the power of the lifestyle or environmental factors in the model was weak because the coefficient of determination was merely 0.070.

### Genetic variant associated with CEA level in healthy populations

There were 25 SNPs associated with CEA levels at the genome wide level based on the analysis using the Multiple Linear Regression model. The biological functions of these SNPs were listed in Table S8 and genes involved (except for rs8111500) were *FUT1, FUT2, FUT3, FUT6, ABO, DBP, CA11, RPL18, SULT2B1,* and *FAM83E.* The genes *FUT1, FUT2, FUT3, FUT6, DBP, CA11, RPL18, FAM83E* are located in regions 19p13.3 and 19q13, whereas ABO is located in 9q34.2. *FUT1, FUT2, FUT3, FUT6* and *ABO* are genes involved in the biosynthesis pathway of ABH and Lewis histo-blood group antigens. The five genes encode glycosyltransferases that catalyze the stepwise addition of specific monosaccharide unit in the biosynthesis process.

The strongest CEA level-associated SNP was rs1047781 at 19q13, a missense change (A->T, Ile->Phe) within the *FUT2* gene that encodes a fucosyltransferase catalyzing the production of the H antigen and determines the secretion status of the ABO and Lewis histo-blood group antigens. It was reported that the T allele of this SNP represents a *de facto* inactivated mutant of the *FUT2* enzyme and was causally associated with the non-secretor phenotype in a Japanese population [30]. But the direct experimental evidences supporting the biology association between various forms of ABO, Lewis histo-blood group antigens and CEA levels are absent. Some studies have provided indirect evidences that the A antigen moiety coded by ABO is present on CEA molecules [31], and that the determinants of A, B and H antigens and of CEA shared the same glycoprotein carrier molecules [32]. Thus it may be speculated that the genetic polymorphisms of various CEA antigen-synthesizing glycosyltransferases may modify the sugar groups of the CEA molecules, leading to varying stabilities of CEA molecules in the blood, or alternatively resulting in different detection rates by the given CEA measurement method.

We found eight SNPs located in the *ABO* gene, encoding an acetyl-galactosaminyltransfer- ase that determines the ABO blood types. Based on the data from Seattle SNPs (http://pga.mbt. washington.edu) as well as the Blood Group Antigen Mutation Database (www.ncbi. nlm.nih.gov), an associated study revealed that rs507666 is a surrogate for type A1 histo-blood group antigen, rs687289 is a marker for the O allele, rs8176746 is a marker for the B allele and rs8176704 is a marker for the A2 allele [33]. This interesting finding might support the association between CEA levels and *ABO* gene in CRC patients; this result is shown in Figure 6 and 7.

Furthermore, the ABO histo-blood group type has been associated with a series of diseases characterized by elevated level of serum CEA, such as Helicobacter pylori infection, cancer of the gastro-intestinal tract, breast cancer, and pulmonary cancer [34], [35]. The ABO blood type group exhibiting a high level sCEA might suggest a high risk for these diseases in the ABO carrier population [36], [37]. Because of the lack of knowledge of CEA molecular synthesis, it is hard to do further bioinformatics analysis in addition to that for *ABO* and *FUT* genes.

The purpose of our phase one study was to find loci to be used to select candidate loci for SNP genotyping in CRC patients. We inferred that SNPs were associated with elevated sCEA levels in healthy and CRC population from the same region. Therefore candidate SNPs were determined based on rank of genome-wide *P*-value and location of genes relevant to ABO blood type. Rs8176746, rs376077, rs12608544, rs3786749, rs2071699, rs441810 and rs507666 were subsequently included in the genotyping of CRC patients.

### Genetic variation associated with serum CEA levels in CRC patients

In order to estimate the heterogeneity, potential non-genetic confounders affecting CEA levels in healthy populations were analyzed in CRC patients at first and revealed that the status of lymph node metastasis was a significant contributor. Stratification analysis showed that rs1047781 (chr19- FUT2) was the associated loci in CRC patients. This suggests that there might be confounders in addition to the status of lymph node metastasis. In accordance with the results from the GWAS analysis in healthy population, the rs1047781 was a susceptible locus for elevated CEA level in CRC patients. In healthy population, the CEA level of AA genotype was the lowest and TT was the highest (data have been adjusted for confounders). Similarly, genotype AA carriers had the lowest CEA levels in the CRC patients without tumor metastasis. The allele T (thymine) to A (adenine) in rs1047781 is a missense mutation. The other study in FAMHES showed rs1047781 to be a locus associated with vitamin B12 levels in healthy people [38]. However, this was not determined in CRC population. So far, there has been no convincing evidence about Vitamin B12 and any other lifestyle factors affecting CRC recurrence and survival [39]. An *in vitro* experiment showed that the CDX2 gene is associated with the increase in sLe^x/a^ expression and suppression of FUT2 in colon cancer cells during EGF/bFGF-induced epithelial–mesenchymal transition (EMT) [40]. EMT is a critical phenotypic alteration in metastatic cancer cells. We observed that AT and TT genotypes of rs1047781 (FUT2), and not the AA genotype, are associated with higher serum CEA levels. Interestingly, the CRC patients with T allele exhibited a different prognosis from those with the AA genotype. The results suggest that CDX2 might be inactive in allele T carriers, resulting in a difference of expression between FUT2 and CEA. The allele T carrier might be susceptible to tumor metastasis at the early stage of CRC and to tumor recurrence in the future. It was reported that β1, 3- Galactosyltransferase is down-regulated in colon adenocarcinomas and causes CEA express with absence of type 1 chain [41], but the relation between FUT2 and CEA was still unknown. Therefore laboratory evidence is needed to prove whether this polymorphism is biologically functional [42].

### Risk factors for preoperative CEA levels and tumor recurrence in CRC patients

The age, cigarette smoking, alcohol drinking and obesity were confounders for the serum CEA levels in healthy people. Among CRC patients, the status of metastasis in regional lymph nodes and distant organs were main confounders of preoperative CEA levels in our study. To test if tumor size is a confounder [43], in our study, the association between tumor size and pre CEA levels was examined yet the association was not significant (see Figure S2). BMI is affected by advancing age more than 20 yrs old and it is not an independent variety in our analysis [44]. Literatures reported that age, obesity, alcohol intake and cigar smoking were independent risk factors for the prognosis of CRC. Age (especially ≥75 yrs.) is the important risk factor for patients with CRC regardless of the type of therapy and grade of tumor stage [45].

For patients who underwent curative resection, the prognosis of elderly patients (≥75 yrs.) was also worse than the younger (25∼75 yrs) [46]. However, we did not find this association in our population. Prospective studies suggested that cigarette smoking is associated with the mortality of CRC [47]. Proposed mechanism of this pathogenesis is through the DNA damage caused by carcinogens of cigar smoke [48], [49]. Obesity and the accompanying chronic systemic inflammation and metabolic alteration are risk factors for CRC [50]. It was reported that gender is also a potential factor affecting the prognosis of CRC but only in patients at age <40 yrs [51]. Thus we considered that it is inclusive and did not take it as a confounder in relevant analysis.

### Genetic variant associated with tumor recurrence in CRC patients

The elevations of CEA levels in healthy and CRC populations might suggest biological relationship between synthesis of ABH antigen and CEA. Although the preoperative CEA levels were associated with ABO allelic genotypes and tumor recurrence, the disease-free times of groups with blood types A and O were not significantly different in survival analysis (Figure S3). This result also suggested that the ABO blood types were not biomarkers of tumor recurrence prediction.

Logistic regression model showed that the preoperative CEA level, age and depth of tumor invasion were risk factors influencing RLN metastasis. Also, the preoperative CEA level was a significant factor contributing to the risk of tumor recurrence in 5 years. It is therefore interesting to explore the association between sCEA-relevant SNPs and lymph node metastasis. As shown in Table 3, rs1047781-TT carriers had lower CEA levels and fewer RLN metastases, supporting the association between CEA levels and prognosis. Since rs8176746 was associated with lymph node metastasis, the combination of rs1047781 and rs8176746 as genetic markers of disease-free survival was applied for further analysis. As suggested by Figure 3, rs1047781 (AT+TT) carriers had worse disease-free survival than the AA carriers. It is in accordance with the higher CEA levels among mutant type rs1047781 carriers.

Although these SNPs might associate with tumor recurrence in CRC, the overall survival of CRC patients was not significantly different in 5 years when grouped by rs1047781 or rs8176746 (Figure S4, S5). This suggests that overall survival of CRC patients was affected by other factors excepted for tumor recurrence. Therefore it is worthwhile to test if rs1047781 is a prognostic marker of tumor recurrence among CRC patients.

Overexpression of the *CEA* gene (and *CEACAM6*) has been found in a series epithelial malignancies including CRC [52], [53]. This could disrupt the colonocyte differentiation and promote colon carcinogenesis [54]. Higher preoperative sCEA levels were associated with higher risk of CRC recurrence after operations. Thus the preoperative sCEA level could be served as an independent prognostic factor [55], [56], [57]. The mutation in the tumor cell's *CEA* gene (PELPK motif; Pro-Glu-Leu-Pro-Lys) might induce high CEA levels by its hepatic clearance; mutation in the PELK motif could alter structural stability and binding affinity to Kupffer's cell receptor in the liver [58]. In addition, this binding results in the release of a series of cytokines that have the potential to activate hepatic sinusoidal endothelium [59]–[61]. These observations imply that the CEA could be a facilitator of hepatic metastasis, although direct association between CEA level and lymph node metastasis is absent.

Another study indentified rs10318 (GREM1), rs6983267 (POU5F1P1, DQ515897, MYC) and rs4464148 (SMAD7) associated with clinic outcome in patients with stage II and stage III CRC treated with 5-FU-based adjuvant chemotherapy [62]. GREM1, BMP2 and SMAD7 are signaling molecules in the TGF-β pathway. Overexpression of TGF-β signaling genes has been found in the progression of CRC [63]. Given the complex process of CRC recurrence and metastasis, this finding suggested there are other genetic risk factors. Interestingly, it was reported that CEA could interact with TGF-β receptor and inhibits TGF-β signaling directly; even enhance the survival of colorectal cancer cells in local colonization in animal study [64].

### Adjuvant therapy of CRC

In our study, some CRC patients with stage II and all the patients with stage III received FOLFOX regiment as adjuvant treatment [65], [66]. However, chemotherapy itself was not associated with tumor relapse in the five years of follow-up. The benefit of adjuvant chemotherapy for patients with stage II CRC is still debatable [67]. As suggested by clinical trials the disease-free survival of 3 years follow-up might be appropriate index in patients with stage II CRC when treated by fluorouracil [68]. Thus the follow-up time was up to 4 years in our study. In a meta-analysis of adjuvant therapy and survival among patients with resected CRC, the longer time to adjuvant therapy was associated with worse survival [69]. It suggested that optimal timing of adjuvant therapy was an important factor and needed to be evaluated in analysis.

## Summary

We reported in this study the genetic variations associated with CEA levels in CRC population. We found that the rs1047781 is the susceptible locus for elevated sCEA level whereas rs8176746 is associated with the status of regional lymph node metastasis. The elevated sCEA level and status of lymph node metastasis are risk factors for tumor-recurrence after radical operation. The SNP rs1047781 might be a predictor for tumor recurrence in TNM stage II and III CRC patients. The contribution of rs8176746 was not addressed in this study.

A number of limitations of our study must be addressed: (1) In addition to CRC, elevated sCEA levels have been reported among cholangio-carcinoma, pancreatic carcinoma and breast cancer patients. Therefore, it is interesting to explore the influence of the candidate SNPs we found in this study in these malignancies. (2) Although candidate SNPs such as rs1047781 is located in the encoding region, its biological function is still unknown based on existing evidence. Therefore experiments exploring the regulatory mechanisms are definitely needed. (3) Because the samples included in our study were limited to Guangxi province, a region in southern China, we have yet to determine the significance of such associated SNPs in populations of other regions.

## Supporting Information

Figure S1 **Result of GWAS—Manhattan plot.**
(TIF)

Figure S2 **Scatter plots of association between tumor size and sCEA levels in CRC patients.** The CRC patients without regional lymph node and distant organ metastasis were included in analysis; tumor size was represented by max tumor diameter.
(TIF)

Figure S3 **Survival analysis for disease-free times of CRC patients grouped by A and O blood type.** The difference of disease-free times was not significantly different (p = 0.391).
(TIF)

Figure S4 **Overall survival of CRC patients grouped by rs8176746.** In rs8176746 (chr-9 ABO), overall survival of CRC patients grouped by allelic genotypes (AA, AG+GG) was not significantly different (p = 0.084).
(TIF)

Figure S5 **Overall survival of CRC patients grouped by rs1047781.** In rs1047781 (chr-19 FUT2), the overall survival of CRC patients grouped by allelic genotypes (AA, AT +TT) was not significantly different (*p* = 0.165).
(TIF)

Table S1 **Demographics of the participants from FangChengGang Area Male Health Examination Survey.** Subjects of Han nationality were included in analyses.
(DOC)

Table S2 **Univariate analysis of factors affecting sCEA levels in FAMHES participants.** CEA values are presented as Mean±S.D. The value of CEA had been log-transformed to fit for normal distribution in analysis.
(DOC)

Table S3 **SNPs associated with sCEA levels in GWA study.** a. Genomic position is based on NCBI build 36. b. a/A: minor allele/major/allele. c. MAF indicate the minor allele frequency for allele a. d. aa indicates serum complement levels for homozygous carriers of minor alleles, Aa indicates for heterozygous carriers, and AA indicates for homozygous carriers of major alleles. e. P values are adjusted for age, folic acid level and BMI.
(DOC)

Table S4 **Baseline Characters of CRC Patients.**
(DOC)

Table S5 **Assay ID of SNPs genotyped in CRC patients.**
(DOC)

Table S6 **Summary of the SNPs genotyping in CRC patients.** There were 194 CRC patients with adenocarcinoma in analysis.
(DOC)

Table S7 **Biological functions of associated SNPs.** Dataset: http://www.ncbi.nlm.nih.gov/projects/SNP/.
(DOC)

Text S1 **Supporting information of stage 1 and stage 2 study.**
(DOC)
