# Supplementary figures and images for: Genetic Variations Affecting Serum Carcinoembryonic Antigen Levels and Status of Regional Lymph Nodes in Patients with Sporadic Colorectal Cancer from Southern China
Source: PLoS One. 2014 Jun 18;9(6):e97923. doi: 10.1371/journal.pone.0097923 (PMC4062418; doi:10.1371/journal.pone.0097923)

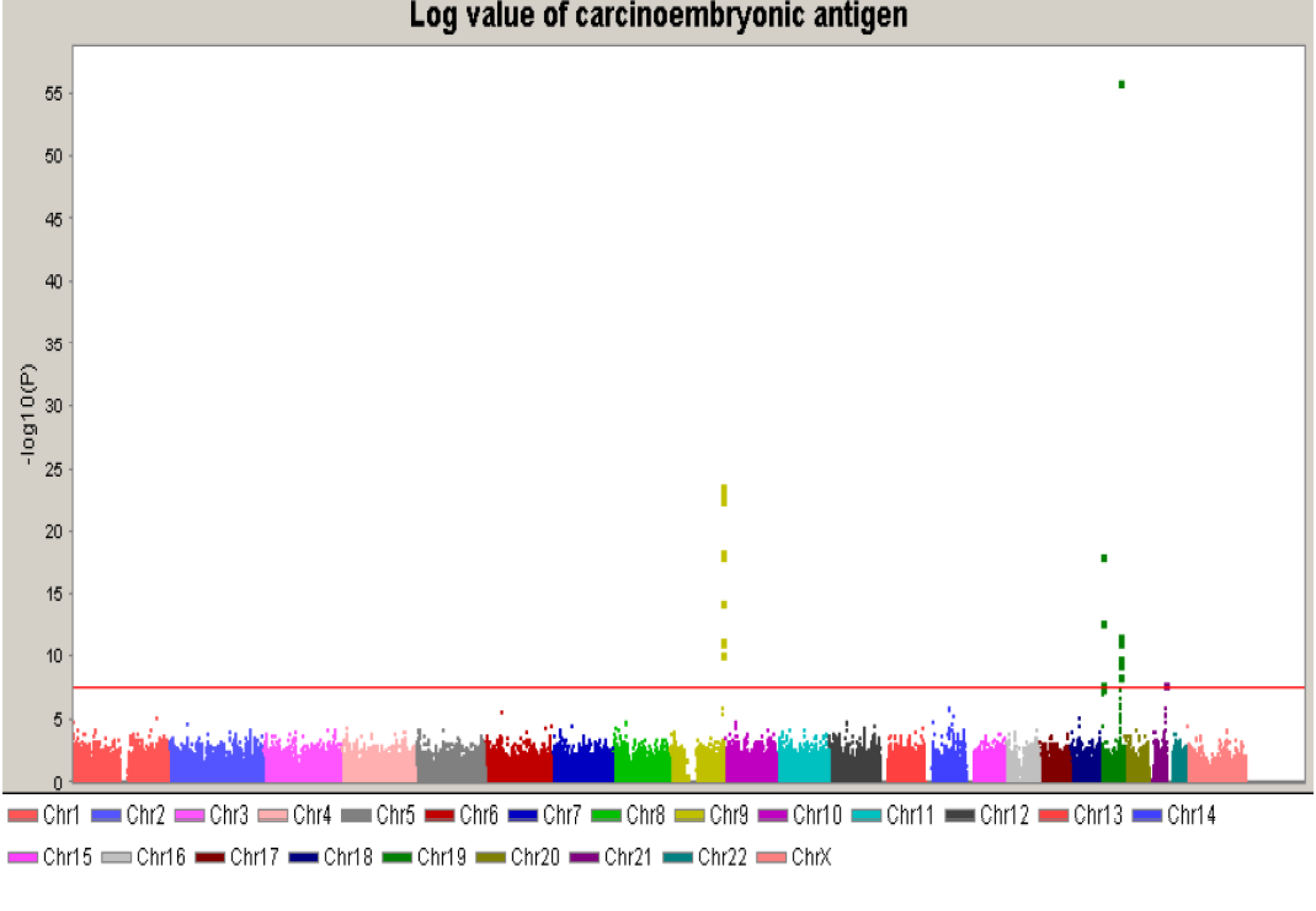

Supplement: Figure S1 — Result of GWAS—Manhattan plot. (TIF) [file pone.0097923.s001.tif]

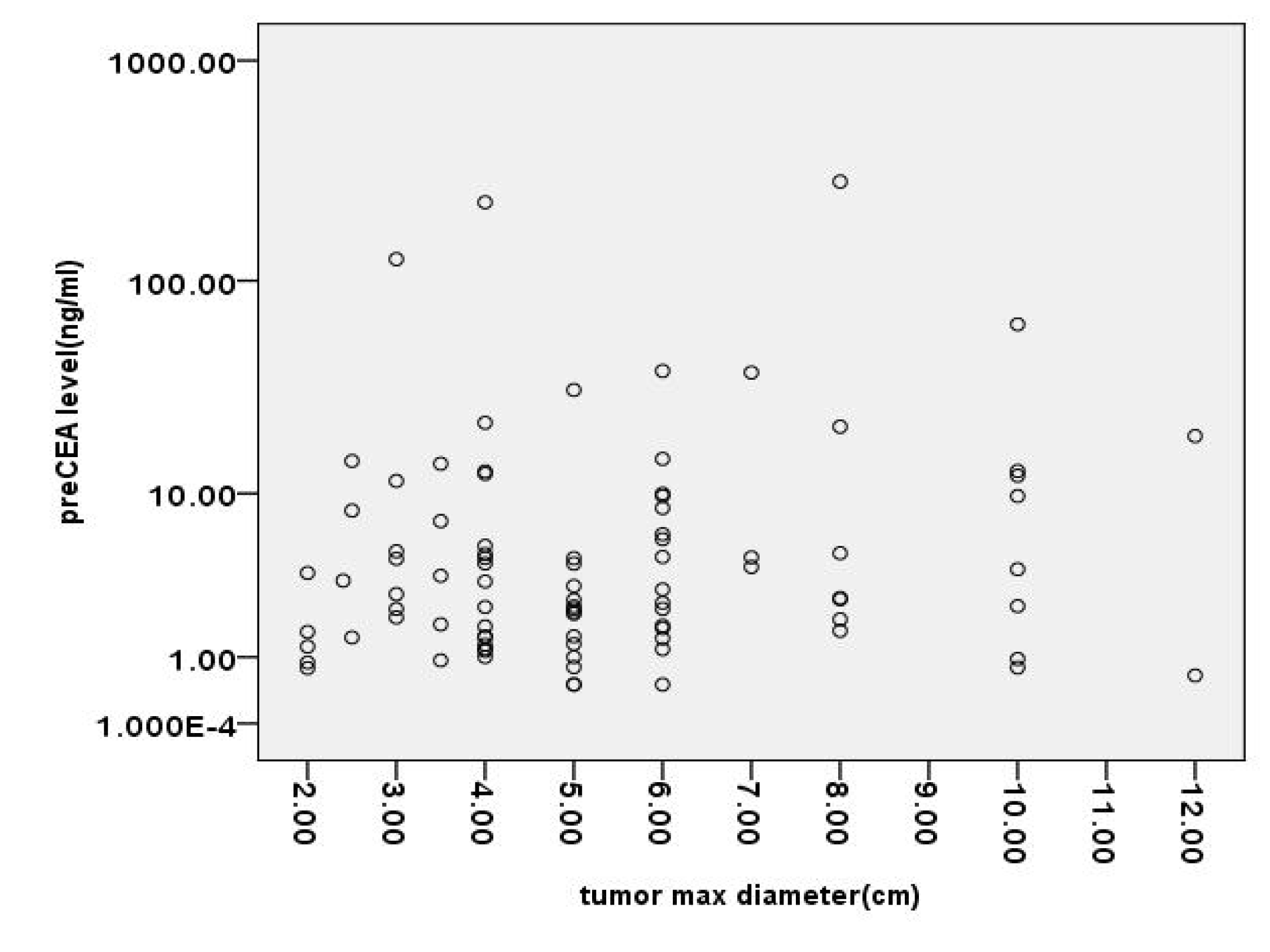

Supplement: Figure S2 — Scatter plots of association between tumor size and sCEA levels in CRC patients. The CRC patients without regional lymph node and distant organ metastasis were included in analysis; tumor size was represented by max tumor diameter. (TIF) [file pone.0097923.s002.tif]

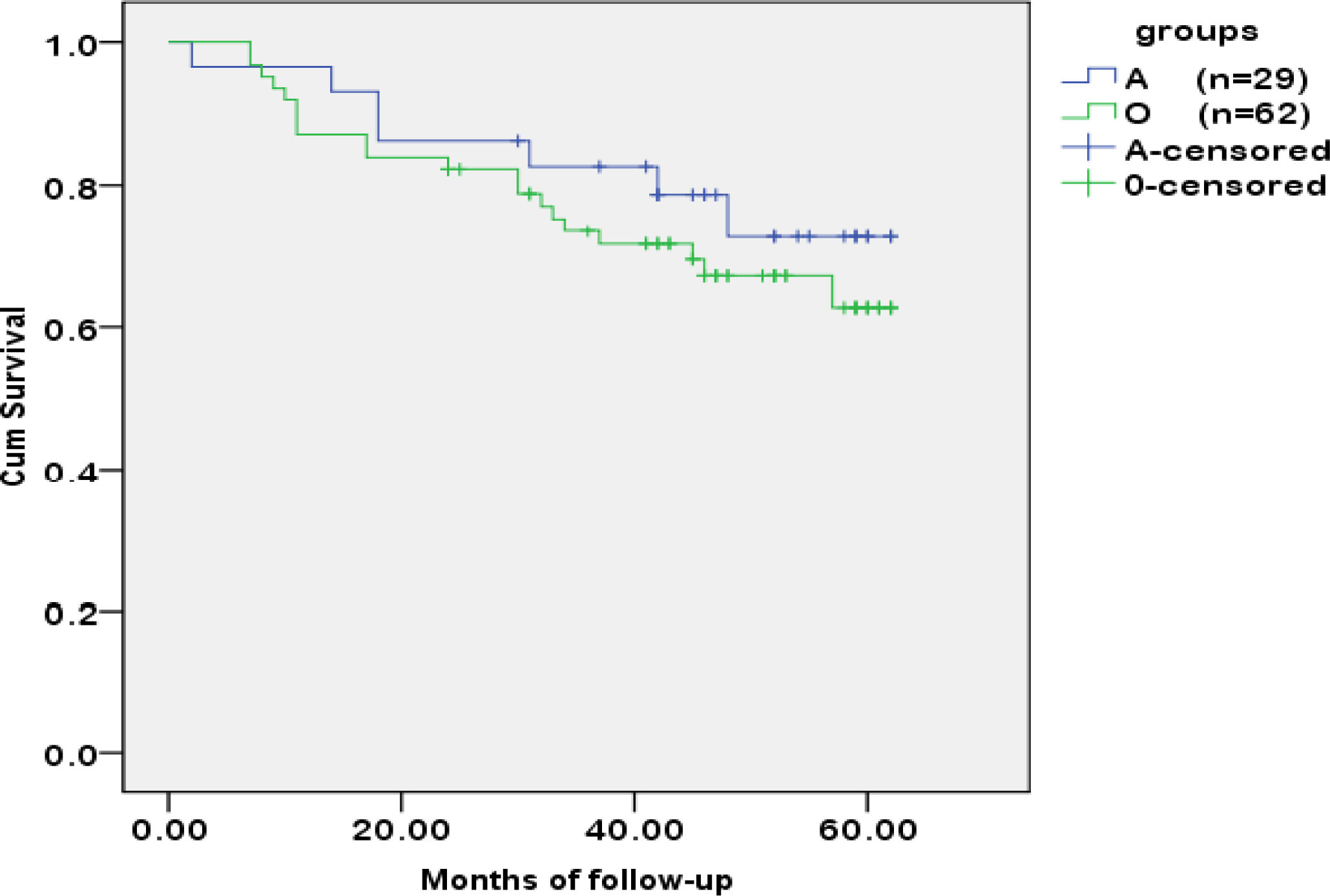

Supplement: Figure S3 — Survival analysis for disease-free times of CRC patients grouped by A and O blood type. The difference of disease-free times was not significantly different (p = 0.391). (TIF) [file pone.0097923.s003.tif]

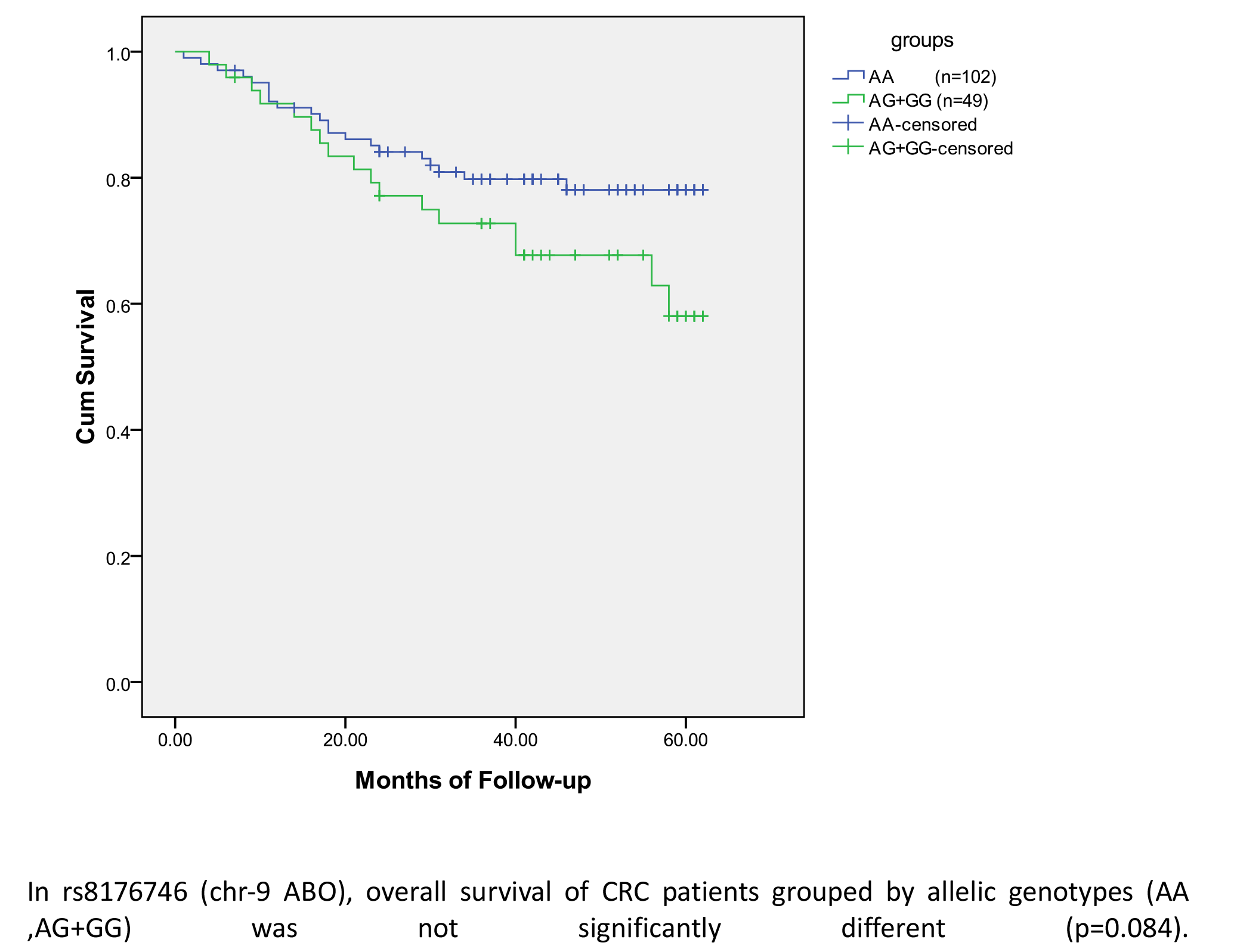

Supplement: Figure S4 — Overall survival of CRC patients grouped by rs8176746. In rs8176746 (chr-9 ABO), overall survival of CRC patients grouped by allelic genotypes (AA, AG+GG) was not significantly different (p = 0.084). (TIF) [file pone.0097923.s004.tif]

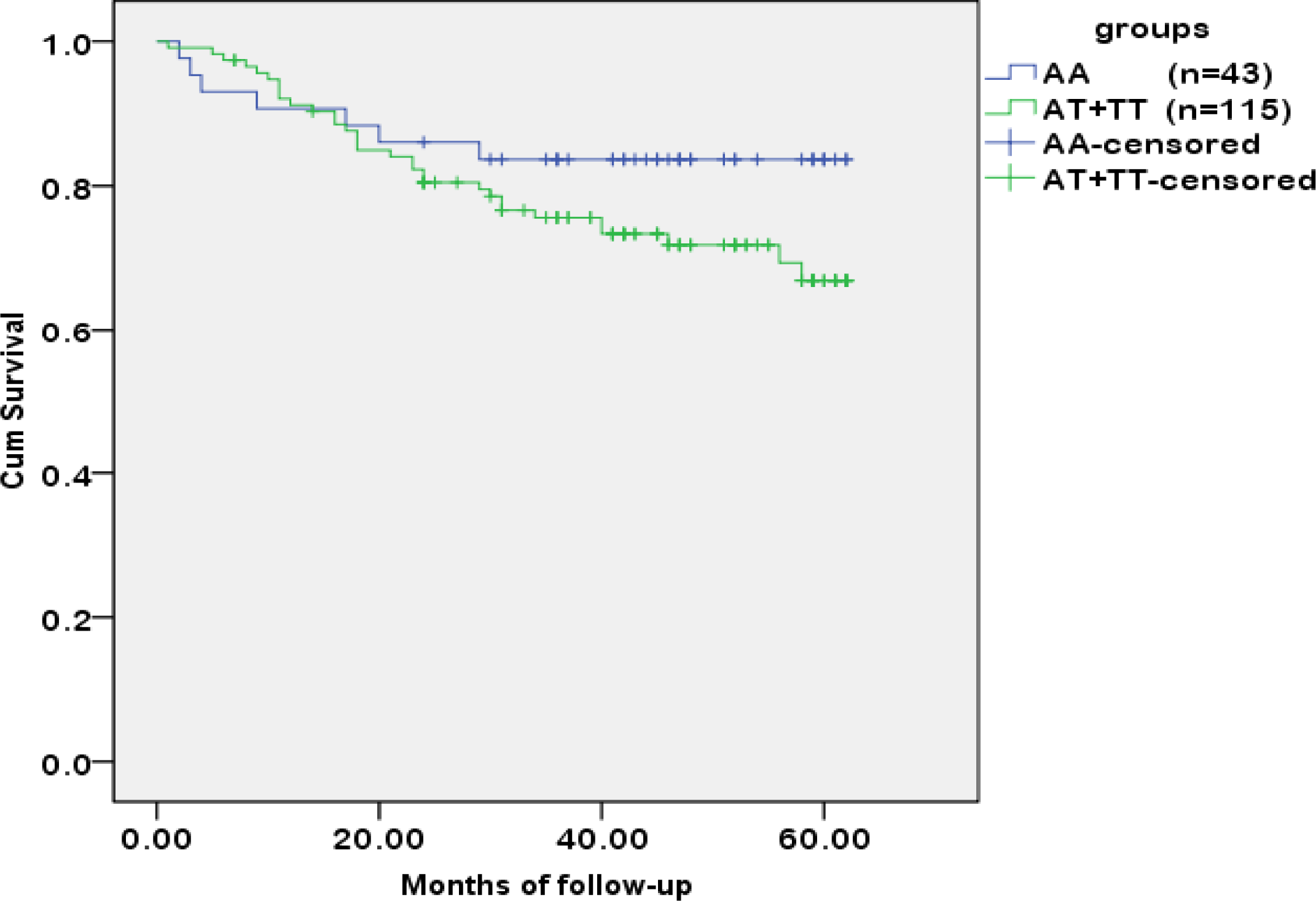

Supplement: Figure S5 — Overall survival of CRC patients grouped by rs1047781. In rs1047781 (chr-19 FUT2), the overall survival of CRC patients grouped by allelic genotypes (AA, AT +TT) was not significantly different (p = 0.165). (TIF) [file pone.0097923.s005.tif]
