# Supplementary material for: Genetic Variations Affecting Serum Carcinoembryonic Antigen Levels and Status of Regional Lymph Nodes in Patients with Sporadic Colorectal Cancer from Southern China
Source: PLoS One. 2014 Jun 18;9(6):e97923. doi: 10.1371/journal.pone.0097923 (PMC4062418; doi:10.1371/journal.pone.0097923)
Supplement: Table S1 — Demographics of the participants from FangChengGang Area Male Health Examination Survey. Subjects of Han nationality were included in analyses. (DOC) [file pone.0097923.s006.doc]

Table S1. Demographics of the participants from FangChengGang Area Male Health Examination Survey

| Variables | N (%) | Values of CEA level (ng/mL) | | | |
| --- | --- | --- | --- | --- | --- |
|  |  | Minimum | Maximum | Mean | S.D |
| Age (yrs) |  |  |  |  |  |
| 17—19 | 0 | - | - | - | - |
| 20—29 | 581(28.7) | 0.4 | 7.3 | 2.1 | 1.14 |
| 30—39 | 664(33.0) | 0.2 | 10.0 | 2.3 | 1.34 |
| 40—49 | 477(23.7) | 0.3 | 9.1 | 2.7 | 1.62 |
| 50—59 | 175(8.7) | 0.6 | 9.2 | 2.8 | 1.60 |
| ≥60 | 115(5.7) | 0.6 | 10.3 | 3.3 | 1.91 |
| BMI |  |  |  |  |  |
| ≤18.4 | 103(5.1) | 0.5 | 7.6 | 2.5 | 1.53 |
| 18.5—23.9 | 1133(56.3) | 0.2 | 9.5 | 2.5 | 1.49 |
| 24—27.9 | 614(30.3) | 0.3 | 10.3 | 2.3 | 1.37 |
| ≥28 | 162(8.1) | 0.5 | 9.2 | 2.4 | 1.39 |
| Smoking |  |  |  |  |  |
| Yes | 1101(54.7) | 0.3 | 10.3 | 2.7 | 1.54 |
| No | 911(45.3) | 0.2 | 9.3 | 2.2 | 1.29 |
| Drinking |  |  |  |  |  |
| Yes | 1728 (85.9) | 0.2. | 10.3 | 2.4 | 1.48 |
| No | 284(17.1) | 0.4 | 9.5 | 2.4 | 1.45 |
| HBsAg |  |  |  |  |  |
| Positive | 211(10.4) | 0.3 | 6.2 | 2.5 | 1.47 |
| Negative | 1795(89.3) | 0.2 | 9.5 | 2.4 | 1.17 |
| Unknown | 6 (0.3) |  |  |  |  |

Subjects of Han nationality were included in analyses.
