# Supplementary material for: Genetic Variations Affecting Serum Carcinoembryonic Antigen Levels and Status of Regional Lymph Nodes in Patients with Sporadic Colorectal Cancer from Southern China
Source: PLoS One. 2014 Jun 18;9(6):e97923. doi: 10.1371/journal.pone.0097923 (PMC4062418; doi:10.1371/journal.pone.0097923)
Supplement: Table S2 — Univariate analysis of factors affecting sCEA levels in FAMHES participants. CEA values are presented as Mean±S.D. The value of CEA had been log-transformed to fit for normal distribution in analysis. (DOC) [file pone.0097923.s007.doc]

Table S2. Univariate analysis of factors affecting sCEA levels in FAMHES participants

|  | N | CEA (ng/ml) | F/t | P-value |
| --- | --- | --- | --- | --- |
| Age（years） |  |  | F=21.824 | <0.001 |
| 20—29 | 581 | 1.27±0.24 |  |  |
| 30—39 | 664 | 1.29±0.26 |  |  |
| 40—49 | 477 | 1.35±0.28 |  |  |
| 50—59 | 175 | 1.40±0.26 |  |  |
| ≥60 | 115 | 1.45±0.26 |  |  |
| Cigarette-Smoking |  |  | t＝8.181 | <0.001 |
| yes | 1101 | 1.4±0.25 |  |  |
| no | 911 | 1.3±0.27 |  |  |
| Grading of smoking |  |  | F=27.486 | <0.001 |
| mild | 384 | 1.30±0.25 |  |  |
| moderate | 367 | 1.35±0.24 |  |  |
| severe | 352 | 1.44±0.25 |  |  |
| Alcohol-drinking |  |  | *t*＝0.956 | 0.294 |
| yes | 1737 | 1.32±0.27 |  |  |
| no | 285 | 1.32±0.25 |  |  |
| BMI |  |  | F=2.89 | 0.034 |
| ＜18.5 | 103 | 1.32±0.25 |  |  |
| 18.5—23.9 | 1133 | 1.33±0.27 |  |  |
| 24—27.9 | 614 | 1.29±0.26 |  |  |
| ≥28 | 162 | 1.33±0.25 |  |  |
| HBsAg |  |  | t＝1.357 | 0.175 |
| Positive | 211 | 1.34±0.23 |  |  |
| negative | 1795 | 1.31±0.27 |  |  |

CEA values are presented as Mean±SD. The value of CEA had been log-transformed to fit for normal distribution in analysis.
