# Supplementary material for: Genetic Variations Affecting Serum Carcinoembryonic Antigen Levels and Status of Regional Lymph Nodes in Patients with Sporadic Colorectal Cancer from Southern China
Source: PLoS One. 2014 Jun 18;9(6):e97923. doi: 10.1371/journal.pone.0097923 (PMC4062418; doi:10.1371/journal.pone.0097923)
Supplement: Table S3 — SNPs associated with sCEA levels in GWA study. a. Genomic position is based on NCBI build 36. b. a/A: minor allele/major/allele. c. MAF indicate the minor allele frequency for allele a. d. aa indicates serum complement levels for homozygous carriers of minor alleles, Aa indicates for heterozygous carriers, and AA indicates for homozygous carriers of major alleles. e. P values are adjusted for age, folic acid level and BMI. (DOC) [file pone.0097923.s008.doc]

**Table S3. SNPs associated with sCEA levels in GWA study**

| SNP | Chr^a^ | Genes | Alelle^b^ | MAF^c^ | N=1,999 | | | |
| --- | --- | --- | --- | --- | --- | --- | --- | --- |
|  |  |  |  |  | Mean levels (ng/ml) | | | |
|  |  |  |  |  | aa^d^ | Aa | AA | p-value |
| rs1047781 | 19cen-qter | FUT2 | A/T | 0.46 | 1.16±0.44 | 0.68±0.57 | 0.54±0.64 | 1.00E-56 |
| rs8176741 | 9q34.2 | ABO | A/G | 0.17 | 1.09±0.53 | 0.93±0.49 | 0.65±0.63 | 2.07E-24 |
| rs8176746 | 9q34.2 | ABO | A/C | 0.17 | 1.07±0.52 | 0.93±0.49 | 0.65±0.63 | 6.10E-24 |
| rs8176722 | 9q34.2 | ABO | A/C | 0.17 | 1.07±0.52 | 0.92±0.49 | 0.65±0.63 | 1.17E-23 |
| rs8176743 | 9q34.2 | ABO | A/G | 0.17 | 1.07±0.52 | 0.93±0.49 | 0.65±0.63 | 1.82E-23 |
| rs8176749 | 9q34.2 | ABO | A/G | 0.17 | 1.05±0.53 | 0.93±0.49 | 0.65±0.63 | 2.59E-23 |
| rs579459 | 9q34.2 | - | G/A | 0.18 | 0.44±0.61 | 0.58±0.49 | 0.82±0.58 | 4.44E-19 |
| rs507666 | 9q34.2 | ABO | A/G | 0.18 | 0.45±0.61 | 0.58±0.63 | 0.82±0.58 | 7.39E-19 |
| rs3760775  rs7030248 | 19p13.3  9 | FUT6  - | A/C | 0.34 | 1.08±0.51 | 0.74±0.63 | 0.65±0.62 | 8.43E-19 |
|  |  |  | G/A | 0.31 | 0.96±0.50 | 0.81±0.59 | 0.63±0.64 | 4.22E-15 |
| rs3760776 | 19p13.3 | FUT6 | A/G | 0.21 | 1.19±0.58 | 0.79±0.57 | 0.68±0.61 | 2.06E-13 |
| rs12608544 | 19 | DBP | A/G | 0.36 | 0.93±0.54 | 0.77±0.58 | 0.64±0.61 | 2.46E-12 |
| rs11880333 | 19q13.3 | CA11 | A/G | 0.36 | 0.92±0.59 | 0.77±0.60 | 0.64±0.61 | 4.97E-12 |
| rs7873522 | 9q34.2 | ABO | G/A | 0.46 | 0.87±0.54 | 0.75±0.60 | 0.61±0.64 | 5.12E-12 |
| rs2292342 | 19q13 | RPL18 | A/G | 0.35 | 0.87±0.58 | 0.77±0.60 | 0.64±0.60 | 6.78E-12 |
| rs8176720 | 9q34.2 | ABO | G/A | 0.46 | 0.93±0.54 | 0.75±0.60 | 0.61±0.65 | 7.38E-12 |
| rs8176725 | 9q34.2 | ABO | A/G | 0.23 | 0.93±0.58 | 0.83±0.60 | 0.67±0.63 | 6.61E-11 |
| rs3786749 | 19q13.3 | SULT2B1 | A/G | 0.30 | 0.98±0.59 | 0.78±0.55 | 0.66±0.60 | 1.39E-10 |
| rs2071699 | 19q13.1-qter | FUT1 | A/G | 0.24 | 0.98±0.53 | 0.80±0.61 | 0.67±0.62 | 3.35E-10 |
| rs8111500 | 19 | - | G/A | 0.43 | 0.62±0.60 | 0.71±0.58 | 0.84±0.60 | 3.70E-09 |
| rs441810 | 21q22.3 | FAM3B | G/A | 0.26 | 0.95±0.46 | 0.79±0.60 | 0.68±0.64 | 1.52E-08 |
| rs778805 | 19p13.3 | FUT6 | G/A | 0.35 | 0.65±0.62 | 0.67±0.58 | 0.83±0.58 | 1.77E-08 |
| rs778809 | 19p13.3 | FUT6 | G/A | 0.36 | 0.65±0.62 | 0.67±0.61 | 0.83±0.59 | 2.16E-08 |
| rs2306969 | 19p13.3 | FUT3 | A/G | 0.08 | 1.16±0.46 | 0.88±0.61 | 0.71±0.61 | 3.04E-08 |
| rs433852 | 19q13.32-q13.33 | FAM83E | A/G | 0.45 | 0.66±0.59 | 0.69±0.60 | 0.86±0.61 | 5.43E-08 |

a. Genomic position is based on NCBI build 36.

b. a/A: minor allele/major/allele

c. MAF indicate the minor allele frequency for allele a.

d. aa indicates serum complement levels for homozygous carriers of minor alleles, Aa indicates for heterozygous carriers, and AA indicates for homozygous carriers of major alleles.

e. P values are adjusted for age, folic acid level and BMI.
