# Supplementary material for: Genetic Variations Affecting Serum Carcinoembryonic Antigen Levels and Status of Regional Lymph Nodes in Patients with Sporadic Colorectal Cancer from Southern China
Source: PLoS One. 2014 Jun 18;9(6):e97923. doi: 10.1371/journal.pone.0097923 (PMC4062418; doi:10.1371/journal.pone.0097923)
Supplement: Table S4 — Baseline Characters of CRC Patients. (DOC) [file pone.0097923.s009.doc]

**Table S4. Baseline Characters of CRC Patients**

| Variables |  | Number of patients |
| --- | --- | --- |
| Age (years.) | ≥60 | 91 |
| range ( 18～93 yrs.) | <60 | 94 |
| Gender | Male | 116 |
|  | Female | 69 |
| Primary Tumor (T) | T1 | 1 |
|  | T2 | 81 |
|  | T3 | 50 |
|  | T4 | 1 |
| Regional Lymph Nodes (N) | N0 | 108 |
|  | N1 | 61 |
|  | N2 | 16 |
| Distant Metastasis (M) | M0 | 174 |
|  | M1 | 16 |
| TNM stage | StageⅠ | 3 |
|  | StageⅡ | 106 |
|  | StageⅢ | 64 |
|  | stage Ⅳ | 16 |
| Tumor differentiation | Well differentiated | 9 |
|  | Moderately differentiated | 153 |
|  | Poorly differentiated | 21 |
|  | Undifferentiated | 7 |
| Location | Colon | 78 |
|  | Rectum | 111 |
| Preoperative CEA(ng/ml) | Negative(<5g/ml) | 112 |
|  | Positive(≥5g/ml) | 78 |
| Type of surgery | radical operation | 179 |
|  | palliative surgery | 11 |
| Chemotherapy | yes | 101 |
|  | no | 85 |
| Result of follow-up | Tumor recurrence | 57 |
|  | Disease-free survival | 118 |
|  | unknown | 19 |
