# Supplementary material for: Genetic Variations Affecting Serum Carcinoembryonic Antigen Levels and Status of Regional Lymph Nodes in Patients with Sporadic Colorectal Cancer from Southern China
Source: PLoS One. 2014 Jun 18;9(6):e97923. doi: 10.1371/journal.pone.0097923 (PMC4062418; doi:10.1371/journal.pone.0097923)
Supplement: Table S5 — Assay ID of SNPs genotyped in CRC patients. (DOC) [file pone.0097923.s010.doc]

**Table S5. Assay ID o**f SNPs genotyped in CRC patients

| SNPs | Assay ID |
| --- | --- |
| rs8176746 | C__25610772_20 |
| rs3760775 | C__27478412_10 |
| rs441810 | C___2265387_10 |
| rs12608544 | C__3098451_10 |
| rs3786749 | AH0IXPY |
| rs1047781 | C__8832449_10 |
| rs2071699 | C__15869610_20 |
| rs507666 | C__997956_10 |
| rs687289 | C__9326428_20 |
