# Supplementary material for: Genetic Variations Affecting Serum Carcinoembryonic Antigen Levels and Status of Regional Lymph Nodes in Patients with Sporadic Colorectal Cancer from Southern China
Source: PLoS One. 2014 Jun 18;9(6):e97923. doi: 10.1371/journal.pone.0097923 (PMC4062418; doi:10.1371/journal.pone.0097923)
Supplement: Table S6 — Summary of the SNPs genotyping in CRC patients. There were 194 CRC patients with adenocarcinoma in analysis. (DOC) [file pone.0097923.s011.doc]

**Table S6.** Summary of the SNPs genotyping in CRC patients

| rsSNP (alleles) | Numbers of genotyping samples | Hardy-Weinberg  Equilibrium test (P-value) | Minor allele frequency (MAF) (%) | genotype call rate  (%) |
| --- | --- | --- | --- | --- |
| rs8176746 (G/T) | 189 | 0.173 | 0.1859 | 0.9317 |
| rs3760775 (T/G) | 199 | 0.290 | 0.3313 | 0.9849 |
| rs441810 (G/A) | 183 | 0.357 | 0.2664 | 0.9195 |
| rs12608544 (A/G) | 196 | 0.399 | 0.3804 | 0.9849 |
| rs3786749 (T/C) | 135 | 0.412 | 0.3596 | 0.6784 |
| rs1047781 (T/A) | 190 | 0.610 | 0.4512 | 0.9548 |
| rs2071699 (A/G) | 199 | 0.441 | 0.25 | 1.0000 |
| rs507666 (A/G) | 170 | ＜0.001 | 0.4429 | 0.8542 |
| rs687289 (A/G) | 192 | 0.2538 | 0.4151 | 0.9648 |

There were 194 CRC patients with adenocarcinoma in analysis.
