# Supplementary material for: Genetic Variations Affecting Serum Carcinoembryonic Antigen Levels and Status of Regional Lymph Nodes in Patients with Sporadic Colorectal Cancer from Southern China
Source: PLoS One. 2014 Jun 18;9(6):e97923. doi: 10.1371/journal.pone.0097923 (PMC4062418; doi:10.1371/journal.pone.0097923)
Supplement: Table S7 — Biological functions of associated SNPs. Dataset: http://www.ncbi.nlm.nih.gov/projects/SNP/. (DOC) [file pone.0097923.s012.doc]

**Table S7. Biological functions of associated SNPs**

| SNP | Genes | Biological functions |
| --- | --- | --- |
| rs1047781 | FUT2 | missense |
| rs8176741 | ABO | cds-synon |
| rs8176746 | ABO | missense |
| rs8176722 | ABO | located in the intron |
| rs8176743 | ABO | missense |
| rs8176749 | ABO | cds-synon |
| rs579459 | - | - |
| rs507666 | ABO | - |
| rs3760775 | FUT6 | nearGene-5 |
| rs7030248 | - | - |
| rs3760776 | FUT6 | nearGene-5 |
| rs12608544 | DBP | located in the intron |
| rs11880333 | CA11 | located in the intron region |
| rs7873522 | ABO | located in the intron region |
| rs2292342 | RPL18 | nearGene-3 |
| rs8176720 | ABO | cds-synon |
| rs8176725 | ABO | located in the intron region |
| rs3786749 | SULT2B1 | located in the intron region |
| rs2071699 | FUT1 | missense |
| rs8111500 | - | located in the intron region |
| rs441810 | FAM3B | located in the intron region |
| rs778805 | FUT6 | missense |
| rs778809 | FUT6 | nearGene-3 |
| rs2306969 | FUT3 | nearGene-5 |
| rs433852 | FAM83E | nearGene-5 |

Dataset : http://www.ncbi.nlm.nih.gov/projects/SNP/
