# Supplementary material for: Genetic Variations Affecting Serum Carcinoembryonic Antigen Levels and Status of Regional Lymph Nodes in Patients with Sporadic Colorectal Cancer from Southern China
Source: PLoS One. 2014 Jun 18;9(6):e97923. doi: 10.1371/journal.pone.0097923 (PMC4062418; doi:10.1371/journal.pone.0097923)
Supplement: Text S1 — Supporting information of stage 1 and stage 2 study. (DOC) [file pone.0097923.s013.doc]

**Phase one study**

Subjects

The Fangchenggang Area Male Health and Examination Survey (FAMHES) was described in detail elsewhere. In brief, all male FAMHES subjects who received routine physical examinations in the Medical Centre of Fangchenggang First People’s Hospital from September 2009 to December 2009 were invited to participate the study (n=4,364). A total of 4303 subjects (98.6%) consented and donated blood samples. Subjects who were self-reported to be free of diabetes mellitus, coronary heart disease, stroke, hyperthyroidism, rheumatoid arthritis, tumors, and impaired hepatic or renal function were eligible for this study. In addition, only self-reported Han Chinese subjects were selected in the first stage to reduce the degree of genetic heterogeneity. Comprehensive health information was collected through physical examination and demographic information was obtained via a standardized questionnaire. The structured questionnaire was filled by trained staffs in field to collect demographic information for every participant. We limited the present investigation on Han males whose intact demographic information as well as the data of serum CEA levels were available. Finally, the GWAS recruited 2,012 eligible men (age 20-69 yrs) from the FAMHES. The study was approved by the Ethics Committee of Guangxi Medical University.

Detection of serum CEA

Overnight (≤8 hours) fasting blood specimens were obtained. Serum CEA were measured with electrochemiluminescence immunoassay on the COBAS 6000 system (E601, HITACHI, Japan) immunoassay analyzer (Roche Diagnostics, GmbH, Mannheim, Germany), with the same batch of reagents. The intra-assay coefficient of variation was 4.8%, for CEA. In addition, an assay was designed to examine whether the CEA level is due to the cross-reaction of added reagent with the CEA antigens and the examinee’s A/B antigens in the measurement of CEA levels. 6 subjects (2 Type A, 2 Type B and 2 Type O) were selected and their CEA levels were re-measured by adding additional anti-A or anti-B sera or Type O sera into the assay. No significant difference of the CEA levels was found between the two measurements (*P*=0.60), suggesting that the measurement is specific to sCEA levels.

Genotyping SNPs in GWAS

The Illumina Omni 1M chip (Illumina, San Diego, USA) was used for the first stage genome-wide association study (GWAS). Among 2012 genotyped subjects, 1999 passed the QC call rate of 95% were included in the final data analysis. For these subjects, a total of 709211 SNPs passed the following QC criteria: *P*>0.001 for Hardy-Weinberg Equilibrium (HWE) test, minor allele frequency (MAF) >0.01 and genotype call rate >95%.

Allelic genotypes of ABO blood types

The *ABO* gene encodes glycosyltransferases that catalyze the addition of either an N-acetylgalactosamine or a galactose to the H antigen and forms the A antigens (A1 and A2) or the B antigen, respectively. Consistent with the genetic association, allelic genotypes of *ABO* histo-blood types (A1, A2, O, and B), could be deduced by four SNPs within the *ABO* gene (rs507666, rs8176704, rs687289, and rs8176746).

Data analysis of demographic information

Statistical analysis was performed using SPSS 17.0 software (SPSS Inc, Chicago, 17th, U.S.A). CEA values were transformed to achieve normal distribution. The difference of CEA values with regard to each confounding factor was assessed using one-way Analysis of Variance (ANOVA) or *t*-test. General linear regression analysis was used to assess the impact of age, BMI and the amount of smoking cigarettes among HBsAg negative subjects. A two-tailed p value <0.05 was considered statistically significant.

Data analysis of GWAS

The Quality Control (QC) criteria to filter SNPs were: *P*<0.001 for the Hardy-Weinberg Equilibrium (HWE) test, minor allele frequency (MAF) <0.01, and genotype call rate <95%. The IMPUTE computer program was then used to infer the genotypes of SNPs (e.g. SNPs catalogued in Hamap Phase II CHB population release #24,) in the genome that were not directly genotyped. A posterior probability of >0.90 was applied to call genotypes that were imputed from IMPUTE software.

A linear regression model was used to analyze the association between serum CEA levels and SNP genotypes. The age, total quantity of smoking cigarette and BMI were covariates in the model and had been adjusted before association study. The PLINK software package was used to perform this statistical analysis. An additive model was assumed for each SNP tested. Log transformations of CEA level were used to ensure a normal distribution. Population stratification was estimated by a principal component approach, as implemented by EIGENSTRAT software. The top two eigens were adjusted as covariates in the linear regression analysis.

**Phase two study**

Genotyping SNPs in CRC patients

The candidated SNPs was genotyped in CRC patients using the TaqMan-MGB Assay (Applied Biosystems, Foster City, CA, USA), which uses two allele-specific TaqMan MGB probes and a PCR primer pair to detect the specific SNP target. The sequence of the primers and probes are available on request and the TaqMan ®assay ID were listed in Table S7. TaqMan® PCR was performed according to the manufacturer’s standard PCR protocol. The reaction mixture of 25 µL contained 20 ng genomic DNA, 12.5 µL of 2× TaqMan Genotyping Master Mix, 0.75 µL of the primers and probes mix and 10 µL of double distilled water. The amplification was performed under the following conditions: 50°C for 2 min, 95°C for 10 min followed by 45 cycles of 95°C for 15 sec, and 60°C for 1 min. Following the manufacturer's instructions, amplifications were conducted in the 96-well ABI StepOne plus＾^TM^ Real Time PCR System (Applied Biosystems, Foster City, CA, USA) and the allelic discrimination were performed using the SDS 2.1 software (Applied Biosystems, Foster City, CA, USA). The genotyping rates were all above 95% in each reaction. Two negative experimental control (water) were included in each reaction plate. In addition, about 10 samples were randomly selected for repetition.
